# Exploration of quality variation and stability of hybrid rice under multi-environments

**DOI:** 10.1007/s11032-024-01442-3

**Published:** 2024-01-15

**Authors:** Rirong Chen, Dongxu Li, Jun Fu, Chenjian Fu, Peng Qin, Xuanwen Zhang, Zhenbiao Sun, Kui He, Liang Li, Wei Zhou, Yingjie Wang, Kai Wang, Xuanming Liu, Yuanzhu Yang

**Affiliations:** 1 Hunan Province Key Laboratory of Plant Functional Genomics and Developmental Regulation, State Key Laboratory of Chemo/Biosensing and Chemometrics, National Center of Technology Innovation for Saline-Alkali Tolerant Rice, College of Biology, Hunan University, Changsha, 410082 Hunan China; 2 State Key Laboratory of Hybrid Rice, Key Laboratory of Southern Rice Innovation & Improvement, Ministry of Agriculture and Rural Affairs, Hunan Engineering Laboratory of Disease and Pest Resistant Rice Breeding, Yuan Longping High-Tech Agriculture Co., Ltd, Changsha, 410128 China; 3 Software Engineering Institute, East China Normal University, Shanghai, 200062 China; 4 College of Life Sciences, Hunan Normal University, Changsha, 410081 Hunan China

**Keywords:** Hybrid rice, Quality, Phenotype plasticity, Stability, Genome-wide association study

## Abstract

Improving quality is an essential goal of rice breeding and production. However, rice quality is not solely determined by genotype, but is also influenced by the environment. Phenotype plasticity refers to the ability of a given genotype to produce different phenotypes under different environmental conditions, which can be a representation of the stability of traits. Seven quality traits of 141 hybrid combinations, deriving from the test-crossing of 7 thermosensitive genic male sterile (TGMS) and 25 restorer lines, were evaluated at 5 trial sites with intermittent sowing of three to five in Southern China. In the Yangtze River Basin, it was observed that delaying the sowing time of hybrid rice combinations leads to an improvement in their overall quality. Twelve parents were identified to have lower plasticity general combing ability (GCA) values with increased ability to produce hybrids with a more stable quality. The parents with superior quality tend to exhibit lower GCA values for plasticity. The genome-wide association study (GWAS) identified 13 and 15 quantitative trait loci (QTLs) associated with phenotype plasticity and BLUP measurement, respectively. Notably, seven QTLs simultaneously affected both phenotype plasticity and BLUP measurement. Two cloned rice quality genes, *ALK* and *GL7*, may be involved in controlling the plasticity of quality traits in hybrid rice. The direction of the genetic effect of the QTL6 (*ALK*) on alkali spreading value (ASV) plasticity varies in different cropping environments. This study provides novel insights into the dynamic genetic basis of quality traits in response to different cropping regions, cultivation practices, and changing climates. These findings establish a foundation for precise breeding and production of stable and high-quality rice.

**Supplementary Information:**

The online version contains supplementary material available at 10.1007/s11032-024-01442-3.

## Introduction

Rice is one of the important staple crops globally, and hybrid rice technology has greatly increased rice production, ensuring global food security. Along with the progression of society and the enhancement of living standards, there has been a gradual increase in the demand for rice quality (Li et al. 2023). Rice quality is also an important and complex trait, including eating and cooking quality, milling quality, apparent quality, and nutritional quality. The quality of rice directly impacts its commercial value and palatability. Improving the quality of hybrid rice has always been the goal pursued by breeders. Over the years, both traditional and modern molecular breeding techniques have been employed to enhance the quality of hybrid rice steadily (Tian et al. 2009; Zhang et al. 2016). However, the quality traits of rice are susceptible to environmental factors such as light, temperature, and humidity, resulting in variations (Liu et al. 2013; Lu et al. 2022). Therefore, how to mitigate the impact of environmental factors on rice quality and enhance its stability is also one of the concerns in improving hybrid rice.

Phenotype plasticity refers to the ability of the same genotype to produce different phenotypes in different environments (Sultan 2000), reflecting the relationship between organisms and their environment, which is widely present in plants (Bradshaw 1965). Phenotype plasticity is related to the adaptability and stability of plants (Chevin et al. 2013; Finlay and Wilkinson 1963). From an evolutionary perspective, varieties with high phenotype plasticity exhibit stronger adaptability to the environment (Des Marais et al. 2013; Bonamour et al. 2019). However, in the context of crop production, plants with lower phenotype plasticity exhibit greater stability. Consequently, implementing techniques to decrease the phenotype plasticity of crops and enhance their stability becomes crucial in enabling the expression of desired traits across a broader range of locations.

Phenotype plasticity is under genetic control and can be targeted for artificial improvement in crop breeding (Gage et al. 2017). To achieve this goal, based on scientific quantification methods for plasticity, efforts have been made to study the genetic architecture of crop plasticity and analyze the QTLs across various crops (Wang et al. 2015a; Kadam et al. 2017; Jin et al. 2023). Exploratory studies on rice have revealed the genetic structure and potential QTLs underlying the plasticity of yield-related traits (Kikuchi et al. 2017; Mu et al. 2022). Although the precise functions of these QTLs remain unclear, they provide new insights and methods for artificially selecting and improving crop phenotype plasticity. Quality-related traits are influenced by the environment, exhibiting phenotype plasticity. However, previous studies on phenotype plasticity in rice have primarily focused on yield-related traits, but there has been limited research on the patterns and genetic architecture of phenotype plasticity in quality traits, which are crucial for breeding advancements. Enhancing rice quality stability has implications for enhancing the potential and commercial value of rice varieties. Therefore, understanding the patterns of phenotype plasticity and genetic structure of quality-related traits in rice will provide better references for breeding improvements.

Parents selection is crucial in hybrid rice breeding (Chen et al. 2019). However, the challenge of selecting ideal parental materials from a large population to induce strong heterosis is significant. Therefore, breeders use the combining ability to assess the breeding value of parental materials in hybrid production (Sprague and Tatum 1942). By identifying the combining ability of parents in phenotype plasticity, breeders can predict the performance of hybrid combinations, thereby enhancing the efficiency and stability of hybrid rice production (Abd El-Aty et al. 2022).

The large-scale phenotypic analysis is an important foundation for plasticity research. In this study, a total of 141 hybrid rice combinations were obtained from 7 TGMS lines and 25 restorer lines. These combinations were planted in five locations in Southern China in the 2020 summer season and arranged 3 to 5 intermittent sowings in each trial location. Seven quality traits and their phenotype plasticity were investigated, including amylose content (AC), alkali spreading value (ASV), gel consistency (GC), chalkiness degree (CD), percentage of grains with chalkiness (PGWC), transparency (TP), and milled rice ratio (MRR). We analyzed the combining ability of phenotype plasticity for 32 parental materials and elucidated the genetic structure of phenotype plasticity for quality-related traits. Genetic effects and candidate gene analyses were conducted on the identified QTLs. The statistical results suggest that delaying sowing date is beneficial for enhancing rice quality in the Yangtze River basin. We utilized a model to evaluate the phenotype plasticity of each hybrid and identify TGMS and restorer lines that exhibit improved quality stability in hybrid rice breeding. Furthermore, our research uncovered the genetic basis of phenotypic plasticity in rice quality traits and discovered QTLs associated with quality plasticity while predicting candidate genes. These findings offer theoretical guidance for determining the optimal sowing date of high-quality rice and enhancing the quality stability of hybrid rice.

## Materials and methods

### Plant materials and field experiments

In this study, a total of 141 hybrid rice combinations were obtained by crossing 7 TGMS lines with 25 restorer lines. The TGMS lines include Jing4155S (J4155S), LongZhen36S (LZ36S), HuaYue468S (HY468S), ZhenXiangS (ZXS), HuaXuan302S (HX302S), LongKe638S (LK638S), and HuaWei338S (HW338S). The restorer lines include HuaHui8612 (HH8612), HuaHui7810 (HH7810), HuaHui3135 (HH3135), HuaHui7503 (HH7503), TaiSi (TS), LongKeSiMiao13 (LKSM13), HuaHui8012 (HH8012), HuaHui2646 (HH2646), HuaHui6210 (HH6210), HuaHui3748 (HH3748), DiZhan (DZ), HuaHui8037 (HH8037), HuaHui5106 (HH5106), HuaHui8549 (HH8549), YuZhan (YZ), HuaHui8700 (HH8700), HuaHui8954(HH8954), HuaHui4153 (HH4153), R1206, R1212, R1377, R1988, WuShanSiMiao (WSSM534), HuaZhan (HZ), and Jin4 (J4). All parental lines are elite parents in hybrid rice production and sourced from the Core Germplasm Bank of Commercial Hybrid Rice Breeding in Yuan Longping High-tech Agriculture Co., Ltd. (Supplementary Table S1, S2).

The field experiments were conducted in the summer season of 2020 in five locations including SC-GH (Guanghan, Sichuan Province, 104°25′ E, 30°99′ N), HN-LS (Lingshui, Hainan Province, 109°45′ E, 18°22′ N), HN-CS (Changsha, Hunan Province, 112°59′ E, 28°12′ N), HB-EZ (Ezhou, Hubei Province, 114°52′ E, 30°23′ N), and AH-HF (Hefei, Anhui Province, 117°17′ E, 31°52′ N). Among the five trial locations, HN-LS is situated in the south China rice cropping region, SC-GH is located in the upper Yangtze River rice cropping region, while AH-HF, HN-CS, and HB-EZ are situated in the middle and lower Yangtze River rice cropping region. In SC-GH, three intermittent sowings were arranged from April 1 to May 1, with a 10-day interval between sowings. In HB-EZ and AH-HF, four intermittent sowings were arranged from April 15 to June 1, with a 15-day interval between sowings. In HN-CS, five intermittent sowings were arranged from April 10 to June 10, with a 15-day interval between sowings. In HN-LS, four intermittent sowings were arranged from June 15 to July 30, with a 15-day interval between sowings (Supplementary Table S3). Seedlings of 5-leaf age were transplanted. Each material was planted in a five-row plot with eight individuals in each row at a spacing of 20 cm × 26.5 cm density in the field and standard management practices throughout the growing period. At maturity, nine uniform plants in the middle of each plot were harvested.

### Parental DNA extraction and whole genome sequencing

After germination of parental seeds, young leaves were collected and immediately flash-frozen in liquid nitrogen, and then the samples were stored at − 80℃ for future use. DNA extraction was performed using the FastPure Plant DNA Isolation Mini Kit (Vazyme, Jiangsu, China). The concentration of the extracted DNA was evaluated using a NanoDrop spectrophotometer (NanoDrop Technologies, Wilmington, DE, USA) and a Qubit 3.0 fluorometer (Life Technologies, Carlsbad, CA, USA). To assess the purity and integrity of the DNA, 1% agarose gel electrophoresis was conducted. For library preparation, a short-read library with a DNA-fragment insert size of 200–400 bp was generated using 1 μg of genomic DNA. The library preparation was carried out following the manufacturer’s instructions using a library preparation kit compatible with DNBSEQ-T7 (BGI, Shenzhen, China). Subsequently, paired-end (PE) sequencing was performed on a DNBSEQ-T7 platform using the PE 150 model.

### Measurement of quality trait

A minimum of 200 g seeds from each accession were used for measuring the seven quality traits, including AC, ASV, GC, CD, PGWC, TP, and MRR. The CD, PGWC, and TP were measured according to NY/T 2334–2013 (Chinese Ministry of Agriculture Standards). CD, PGWC, and TP were measured with a Microtek Scan Wizard EZ scanner and rice quality analyzer SC-E software (Hangzhou Wanshen Detection Technology Co., Ltd., Hangzhou, China). The AC, GC, and ASV were measured according to Chinese National Standards GB/T 15683–2008(GB/T 22294–2008 and NY/T 83–2017), respectively.

### Measurement of overall quality and meteorological condition

To evaluate the overall quality of the hybrids, we followed the quality standards for cooking rice varieties set by the Chinese Ministry of Agriculture and Rural Affairs (NY/T 593–2021). Here, a *record* is specified as the quality performance of a specific hybrid, which has been planted at a trial location on a particular sowing date. We assessed the quality of all 2774 records in our experiment by applying the standard to each of them.

In order to analyze the correlation between quality characteristics and meteorological conditions, we have also summarized the meteorological data collected at different times within each day into the following meteorological factors: minimum temperature, maximum temperature, average temperature, day/night temperature difference, surface solar radiation, accumulated rainfall, and number of rainy days (Cheng and Zhu 1998). In our analysis, we consider a day with more than 1 mm of rainfall as a rainy day. Spearman correlation was conducted between each quality trait and meteorological factors using the SciPy library in Python.

### Analysis of phenotype plasticity

According to the reaction norm model, the phenotypic record ${y}_{ij}$ of the $i$ th hybrid line observed under the $j$ th environment can be modeled as follows:

1 $${y}_{ij}=\mu +{g}_{i}+{h}_{j}+{b}_{i}{h}_{j}+{\epsilon }_{ij},$$

where $\mu$ is the mean value of the trait, ${g}_{i}$ is the main effect of the $i$ th line, and ${h}_{j}$ is the main effect of the $j$ th environment. ${b}_{i}{h}_{j}$ represents the interaction term (G-by-E) between $i$ th hybrid line (genotype) and $j$ th environment. ${\epsilon }_{ij}$ is the error term.

Here, we introduced the Finlay–Wilkinson regression (FW) as the representation of phenotype plasticity. FW reorganized Eq. 1 into follows:

2 $${y}_{ij}=\mu +{g}_{i}+\left(1+{b}_{i}\right){h}_{j}+{\epsilon }_{ij}.$$

If we consider ${h}_{j}$ as the independent variable of the function${y}_{ij}=f({h}_{j})$, then the slope of this function $(1+{b}_{i })$ indicates the expected trait variation ($\Delta {y}_{i}$) in traits with changes in unit environmental effects ($\Delta {h}_{j}=1$), capable of indicating the phenotype plasticity.

To calculate the plasticity of quality traits at different locations and sowing stages, we selected the hybrid lines with multi-location field trials and at least three intermittent sowings per location from the 141 hybrids. We used the FW package implemented in R by Lian et al. (Lian and De Los Campos 2016) and applied the Bayesian method for regression.

### Analysis of combining ability

Combining ability analysis is a statistical technique employed in plant breeding to assess the genetic potential of parents and predict the performance of their hybrids. The analysis considers both general combining ability (GCA), which measures the average performance of a parent across different crosses, and specific combining ability (SCA), which measures the performance of a specific parent in specific crosses. Since there are no extra replications for each sowing management in our experiment, we could not properly evaluate the SCA for each hybrid. For convenience, we simply treated the SCA as 0. Consequently, the phenotype plasticity of hybrid ${p}_{ij}$, which has parents *i* and *j*, can be modeled as follows:

3 $${p}_{ij}=\mu +{GCA}_{i}+{GCA}_{j}+\epsilon ,$$

where $\mu$ is the mean value of the phenotype plasticity, $\epsilon$ is the error term, and ${GCA}_{i}$ represents the breeding value of phenotype plasticity for $i$ th parent. Since the distribution of ${p}_{ij}$ has a mean value of $\mu$, the sum of the GCAs of all parents is 0 (i.e., ${\sum }_{i}{GCA}_{i}=0$). We used the sommer package (Covarrubias-Pazaran 2016) in R to calculate the GCA in a mixed model, in which the parents and crosses are considered as random effects. If there are no significant additive effects detected from the parents for the plasticity of hybrid traits, the model will indicate a GCA of 0, which will be later displayed in the paper.

### Analysis of BLUP

The analysis for various traits of each location is conducted by fitting a linear mixed model and computing best linear unbiased predictors (BLUPs) with the *lme4* R package (Bates et al. 2015):

4 $$Y=\left(1|LINE\right)+\left(1|ENV\right)+\left(1|LINE:ENV\right),$$

where *Y* represents the phenotypic records, the parentheses indicate random effects, “1|” denotes groups, and “:” refers to interactions. LINE indicates to the hybrid lines, and ENV indicates to different sowing stages or different locations. We utilized the fitted random effects of hybrid lines as the representation of the overall genetic effects of each genotype for these traits while eliminating the environmental effects.

### Variant detection and annotation

The sequencing data was aligned to the *Oryza sativa* Nipponbare reference genome (IRGSP1.0) using BWA mem (Li 2013). PCR and optical duplicates were removed using Picard software (Broad Institute). SNP and InDel detection were performed using GATK HaplotypeCaller (McKenna et al. 2010). To minimize false positives, SNPs that did not meet the following criteria were filtered out: QUAL < 30.0, QD < 2.0, SOR > 3.0, FS > 60.0, MQ < 40.0, MQRankSum < 12.5, or ReadPosRankSum < 8.0. Similarly, InDels that did not meet the following criteria were filtered out: QUAL < 30.0, QD < 2.0, FS > 200.0, MQ < 40.0, or ReadPosRankSum < 20.0. SNPs and InDels were annotated using the software SnpEff (Cingolani et al. 2012) based on their impact on genes. The annotation categorized them into different types, such as synonymous mutations, non-synonymous mutations, frameshift mutations, and others. SnpEff utilized Ensembl release 41 as the gene annotation version for this analysis.

### Genome-wide association analysis

The GEMMA (Zhou and Stephens 2014) software was utilized to conduct Genome-Wide Association Studies (GWAS) for BLUP and FW values of different traits and locations, with a mixed linear model (MLM) fitted. The kinship matrix was used as a random effect, while the first three principal components from principal component analysis (PCA) were included as fixed effects in the MLM. PCA analysis was performed using the smartPCA program implemented in the Eigensoft package (Patterson et al. 2006). The significance threshold for GWAS analysis was determined using the Bonferroni correction method, which means dividing 0.05 by the number of SNPs (n) in the analysis. Here, the threshold was set at *p* < 5.32 × 10^−8^ (i.e., − log10(*p*) < 7.27). The entire genome was partitioned into LD blocks based on an LD (linkage disequilibrium) threshold of *r*^2^ = 0.6 using the gpart R package (Kim et al. 2019). LD blocks with a distance of less than 1 Mb are merged. LD blocks that contain significant SNPs were considered as candidate QTLs.

## Results

### Effect of sowing date and meteorological factors on quality of hybrid rice

**Fig. 1:**
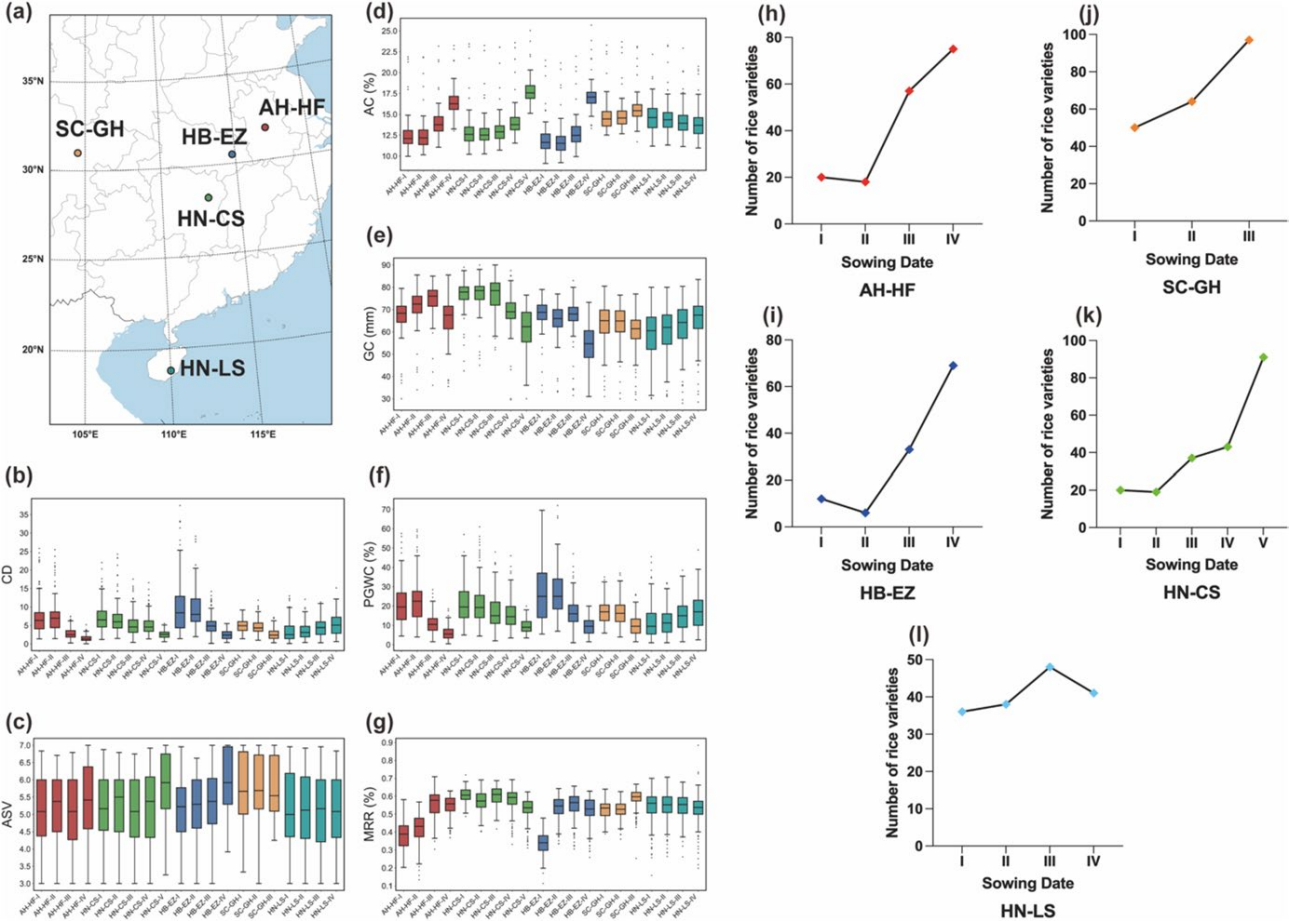
Variation in the trait of hybrid rice combinations across different locations and sowing stages in the southern rice region of China. **a** The five surveyed sites span China’s southern rice region, where 7 quality traits were phenotyped for hybrid rice varieties. **b–g** Boxplot of seven quality traits including **b** chalkiness degree (CD), **c** alkali spreading value (ASV), **d** amylose content (AC), **e** gel consistency (GC), **f** percentage of grains with chalkiness (PGWC), and **g** milled rice ratio (MRR) measured at the five sites in different sowing stages. **h–j** The number of rice varieties reached quality grade 3 or superior quality according to NY/T 593–2021 at each trial location on different sowing dates

The rice quality traits of the hybrids collected from 5 trial locations, with multiple intermittent sowing arrangements, were measured, and the distributions of the trait values are illustrated in Fig. 1a and Supplementary Table S4. Nearly all quality traits in these rice cropping regions exhibited significant correlations with the sowing date, suggesting a general contribution of sowing date to rice quality trait variation (Supplementary Table S5). As sowing dates were delayed, we observed the mean of AC increasing by 2.37–3.97% in AH-HF, 3.82–5.12% in HN-CS, 4.37–5.32% in HB-EZ, and 0.77–0.89% in SC-GH, while decreasing 0.32–0.75% in HN-LS. In contrast, the mean of CD decreased by 1.31–6.2 in AH-HF, 2.71–5.03 in HN-CS, 2.85–7.82 in HB-EZ, and 2.34–2.07 in SC-GH, while increasing by 0.84–1.89 in HN-LS in tandem with the delay in sowing dates (Fig. 1b–g).

We assessed the overall quality of the hybrids by referring to the Chinese Ministry of Agriculture and Rural Affairs’s quality standard for cooking rice variety (NY/T 593–2021). Out of the 2774 records examined, 872 of them exhibited high-quality with grade 3 or superior performance. These high-quality records are sourced from 124 hybrid varieties and shown in all five trial locations and all sowing dates. For all trial locations except HN-LS, the improvement of rice quality correlates with the postponement of sowing dates, with a greater number of records achieving quality grade 3 or higher grades (Fig. 1h–l).

**Fig. 2:**
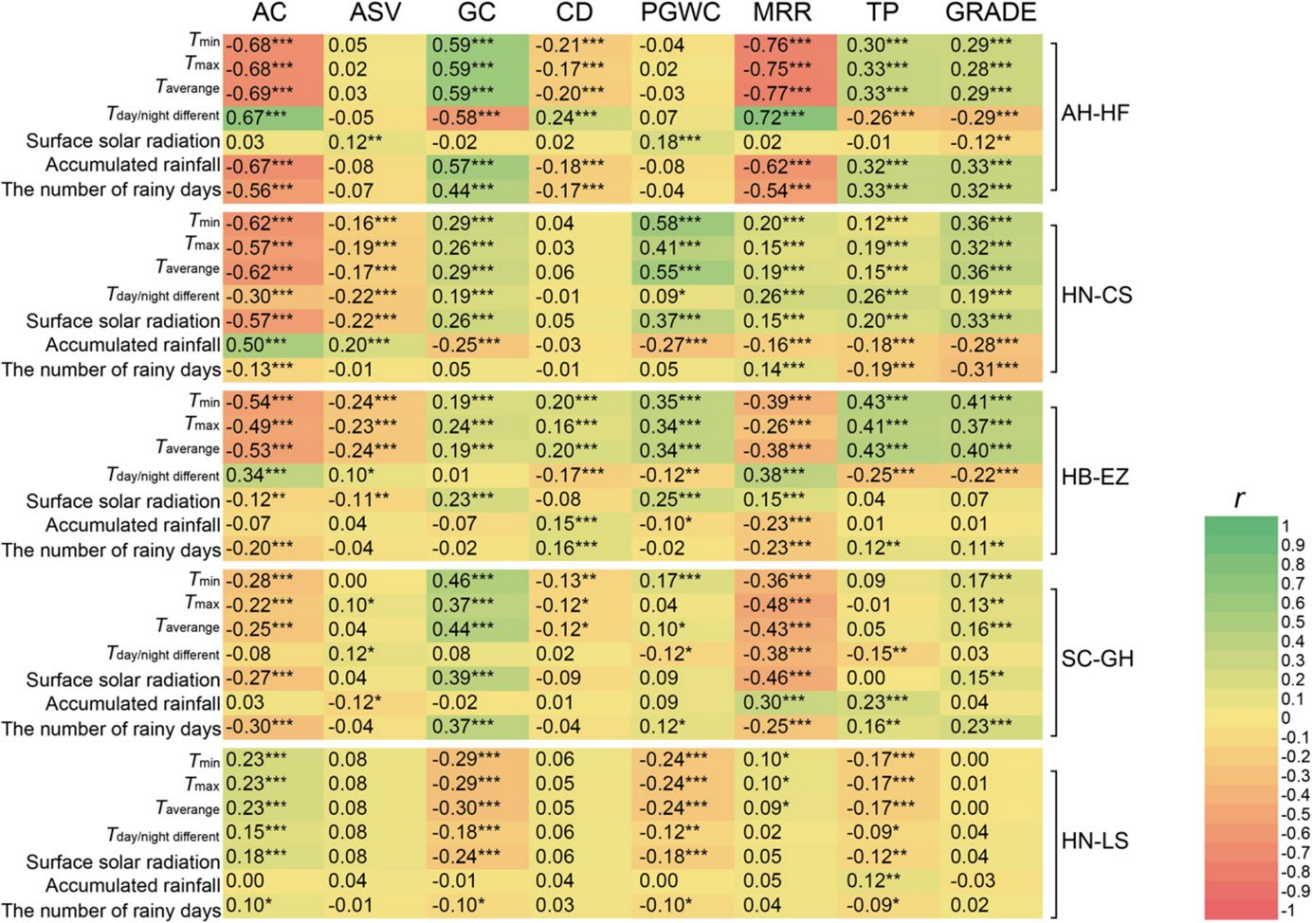
The heatmap illustrates Spearman’s correlation coefficients between meteorological factors and quality traits across five trial locations. *T* denotes temperature. The green color of each box indicates positive relationships between meteorological factors and the corresponding quality trait, while the red color indicates negative relationships. Significant correlations are denoted by asterisks, as determined by a two-tailed *t*-test (**p < 0.001; ***p < 0.0001). *r* stands for correlation coefficients

To assess the meteorological variation more precisely, we decomposed it into the following meteorological factors: minimum temperature, maximum temperature, average temperature, day/night temperature difference, surface solar radiation, accumulated rainfall, and number of rainy days (Cheng and Zhu 1998) (Supplementary Fig. S1). Existing studies have shown that the meteorological factors during the grain filling stage, particularly the initial 15 days following full heading, have a considerable influence on rice quality (Wu et al. 2016; Yan et al. 2021). Thus, we employed the mean values of the aforementioned meteorological factors during this time period for further evaluation*.* We performed an analysis to investigate the correlation between meteorological factors and quality traits. Due to the non-normal distribution of the quality traits data, we computed Spearman correlation coefficients between meteorological factors and these quality traits (Fig. 2). Nearly all quality traits displayed a significant correlation with multiple meteorological factors in each trial location. The correlation trend of each meteorological factor with each quality trait differed across different trial locations. More specifically, at HN-LS, the minimum, maximum, and average temperatures exhibit significant positive and negative correlations with AC and GC, respectively. At the other four locations, there were significant negative and positive correlations between these meteorological factors and AC and GC, respectively. Likewise, at HN-LS, surface solar radiation was positively correlated with AC and negatively correlated with GC. However, at the other four trial locations, contrary correlation trends were observed. In terms of rainy days and accumulated rainfall, our research suggests that increased rainy days are potentially associated with lower levels of AC and higher levels of CD. The significant impact of accumulated rainfall on quality traits was observed, but a more general pattern was not observed in our experiment. Moreover, we considered the rice quality grade as a trait and investigated its correlation with meteorological factors as a reference. For trial locations in the rice cropping region of the upper, middle, and lower reaches of the Yangtze River (i.e., SC-GH, AH-HF, HN-CS, and HB-EZ), there exists a significant correlation between better rice quality and the minimum, maximum, and average temperatures.

### General combining ability analysis for phenotype plasticity of quality traits

**Fig. 3:**
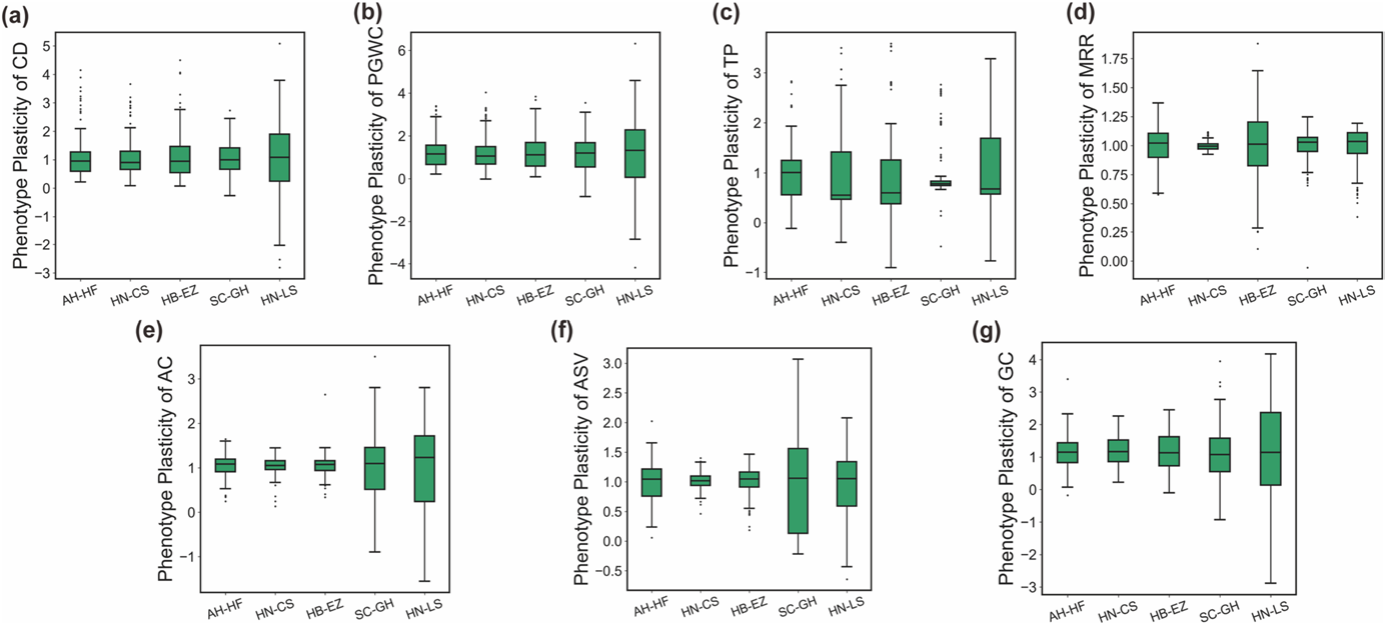
The boxplot illustrates the phenotype plasticity of hybrid rice across five trial locations for the following traits: **a** chalkiness degree (CD), **b** percentage of grains with chalkiness (PGWC), **c** transparency (TP), **d** milled rice ratio (MRR), **e** amylose content (AC), **f** alkali spreading value (ASV), **g** gel consistency (GC)

**Fig. 4:**
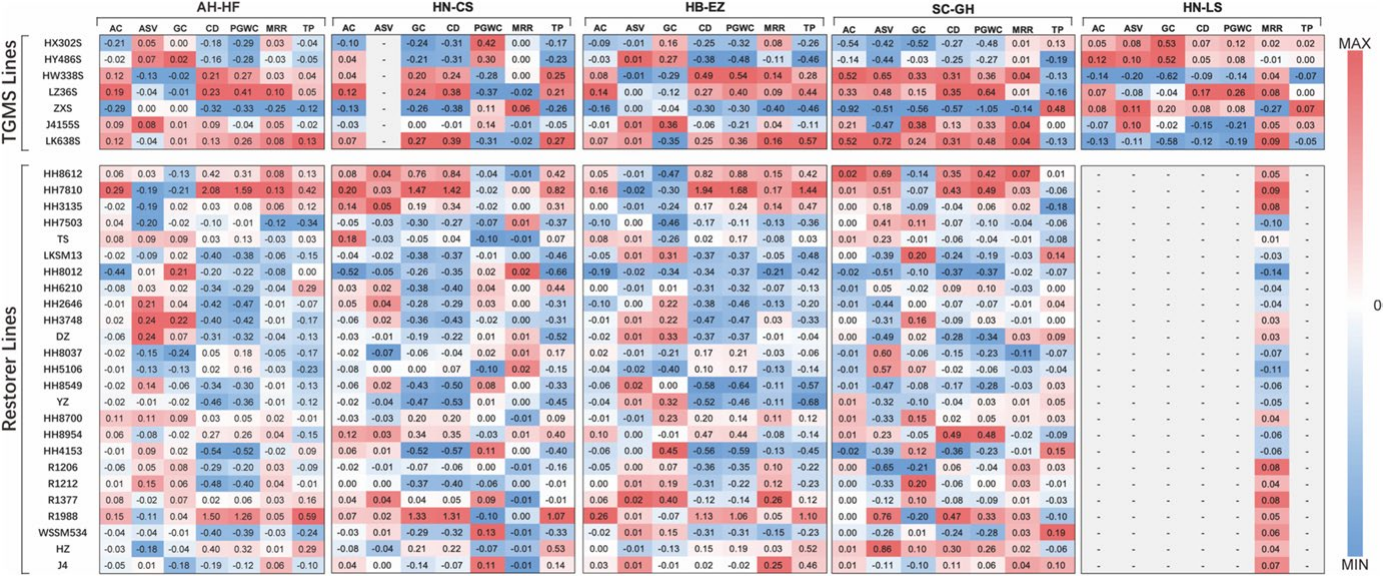
The heatmap illustrates the GCA value for phenotype plasticity in quality traits, as observed between TGMS and restorer lines across various trial locations. Each grid number represents the GCA value for the trait. The red grid denotes a positive GCA value, the blue grid denotes a negative GCA value, and the white grid denotes a zero GCA value

In this study, we evaluated the stability of quality traits for 141 hybrid combinations across five trial locations using the Finlay-Wilkinson regression (FW), a measurement of phenotypic plasticity (Supplementary Table S6) (Fig. 3). The combining ability for phenotypic plasticity was conducted to identify potential parents capable of producing hybrid combinations with stable quality traits. Figure 4 shows the GCA value of the hybrid parents in relation to the plasticity of the seven quality traits. Notably, parents with a low GCA value for plasticity are more likely to derive hybrid combinations with stable quality performance.

Three TGMS lines (ZXS, HX302S, and HY468S) and nine restorer lines (HH8012, HH7503, DZ, LKSM13, HH2646, HH5106, HH8549, YZ, and WSSM534) consistently exhibited comprehensive low GCA values (≤ 0) for plasticity across more than three trial locations. It indicates their potential to consistently produce hybrid combinations with stable quality traits across different cropping regions and varied sowing management. Interestingly, parents with superior quality tend to exhibit low GCA values for plasticity. The 12 parental lines with low plasticity GCA value are all of high quality and have been widely used in high-quality hybrid rice development. We analyzed the quality statistics from yield testing trials of nationally approved hybrid rice varieties derived from all tested parental lines from 2016 to 2022. Notably, the hybrid rice varieties derived from parents with low plasticity GCA values exhibited a higher rate of high quality compared to those derived from parents with high plasticity GCA values (Supplementary Table S7).

### Genome-wide association analysis for phenotype plasticity and BLUP measurement of quality traits

**Fig. 5:**
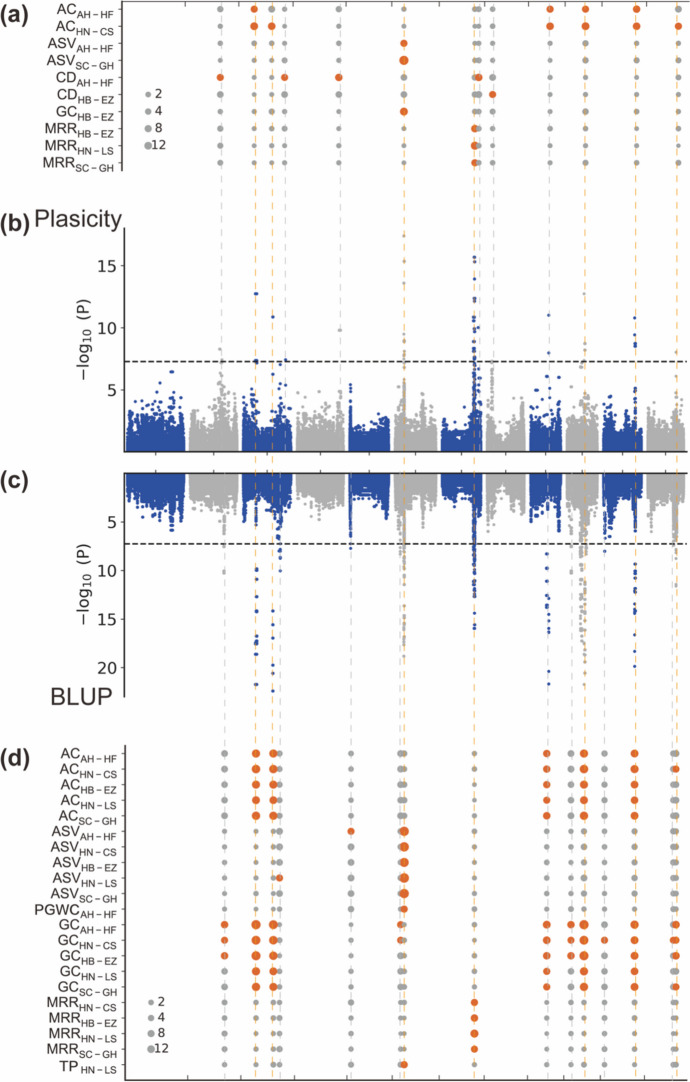
QTLs associated with plasticity and BLUP for seven traits across five trial locations. **a** QTLs associated with the plasticity of different traits across different locations. Each dot represents an SNP, and the size of the dot is proportional to its − log_10_(*p*)-value, as indicated on the right. Loci with a − log_10_(*p*)-value exceeding the genome-wide significance threshold are highlighted in red. **b** Manhattan plot overlays GWAS results of plasticity for the seven traits across five locations. The black horizontal dashed line indicates Bonferroni-corrected genome-wide significance, and the vertical gray lines indicate the positions of detected QTLs. **c** Manhattan plot overlays GWAS results of BLUP for the seven traits across five locations. The vertical red lines indicate the positions of detected QTLs that overlap in both plasticity and BLUP. **d** QTLs associated with the BLUP of different traits across different locations

The genome-wide association study (GWAS) was conducted independently for the plasticity of each quality trait across different trial locations. This analysis revealed a total of 13 QTLs associated with grain quality plasticity (Fig. 5a–b; Table 1). Among these, four QTLs were detected in two trial locations, two QTLs were detected in three trial locations, and the remaining seven QTLs were only detected in a single trial location. These findings underscore the complex genetic basis of plasticity for quality traits in different regions.

**Table 1:** The QTLs associated with plasticity of quality traits in different environments and summary of candidate genes

Loci	Chr	Lead SNP position (bp)	Allele	Candidate region	Trait	Location	− log_10_(*p*)	PVE (%)	Candidate gene	Gene ID	Phenotype
QTL1	2	22,851,743	A/G	22,541,165–24,291,176	CD	AH-HF	8.28536	33.27			
QTL2	3	9,197,833	T/G	8,236,624–10,186,100	AC	AH-HF	7.37350	43.46			
						HN-CS	12.75761	28.99			
QTL3	3	22,570,447	A/G	22,496,257–22,702,315	AC	HN-CS	10.88641	0.74			
QTL4	3	32,401,540	A/T	32,347,009–32430702	CD	AH-HF	7.42889	1.28			
QTL5	4	32,365,134	T/G	32,142,954–33,341,341	CD	AH-HF	9.80603	53.43			
QTL6	6	6,722,905	C/T	6,631,971–7,110,522	ASV	AH-HF	7.83381	28.60	*ALK*	*Os06g0229800*	Grain quality
						SC-GH	31.02958	84.47			
					GC	HB-EZ	13.60619	41.02			
QTL7	7	24,665,290	A/G	23,799,472–25,003,889	MRR	HB-EZ	9.92897	49.79	*GW7|GL7*	*Os07g0603300*	Grain length
						HN-LS	15.69229	68.62			
						SC-GH	7.66602	36.73			
QTL8	7	27,630,430	C/T	27,432,243–28,046,687	CD	AH-HF	10.0137	4.99			
QTL9	8	3,576,864	A/C	3,331,990–3892495	CD	HB-EZ	7.31587	23.53			
QTL10	9	13,969,434	C/T	13,953,334–13,970,114	AC	AH-HF	7.96909	10.02			
						HN-CS	11.01237	0.06			
QTL11	10	13,069,201	A/T	13,064,394–15251405	AC	AH-HF	7.37350	43.46			
						HN-CS	12.75761	28.99			
QTL12	11	23,870,356	A/C	23,842,767–24,454,199	AC	AH-HF	9.4461	17.50			
						HN-CS	10.80975	9.03			
QTL13	12	21,795,764	A/C	21,340,148–21930422	AC	HN-CS	8.03519	9.61			

*PVE* phenotypic variation explained

In the case of AC plasticity, we identified six QTLs were identified on chromosomes 3, 9, 10, 11, and 12. Among these QTLs, four were identified in both HN-CS and AN-HF, while two were exclusively identified in HN-CS. As for CD plasticity, we detected five QTLs were detected on chromosomes 2, 3, 4, 7, and 8. Notably, all QTLs were identified in a single trial location, either HB-EZ or AN-HF.

We identified QTL6 on chromosome 6 as being associated with the plasticity of two key taste quality traits (ASV and GC). The effect of QTL6 on ASV plasticity was detected in both AH-HF and SC-GH, while its effect on GC plasticity was observed exclusively in HB-EZ. These findings suggest that the plasticity of ASV and GC may share a similar genetic basis.

A single QTL, QTL7, located on chromosome 7, was found to be associated with the plasticity of MRR. This association was observed across three trial locations including HB-EZ, HN-CS, and SC-GH.

Furthermore, we employed the best linear unbiased prediction (BLUP) approach to mitigate the impact of non-genetic factors and calculate the genetic effect values for these quality traits (Supplementary Table S8). We also conducted a GWAS for the BLUP measurement. This analysis identified a total of 15 QTLs for the BLUP measurement of seven quality traits across five trial locations (Fig. 5c–d, Supplementary Table S9). Among these QTLs, seven QTLs (QTL2, QTL3, QTL6, QTL7, QTL11, QTL12, and QTL13) were also identified in the previous GWAS of plasticity (Supplementary Table S10). These overlapping QTLs, identified in both GWAS results, could play a crucial role in regulating quality traits and responding to the quality plasticity observed in diverse cropping environments.

### Analysis of QTLs genetic effects and prediction of candidate genes

**Fig. 6:**
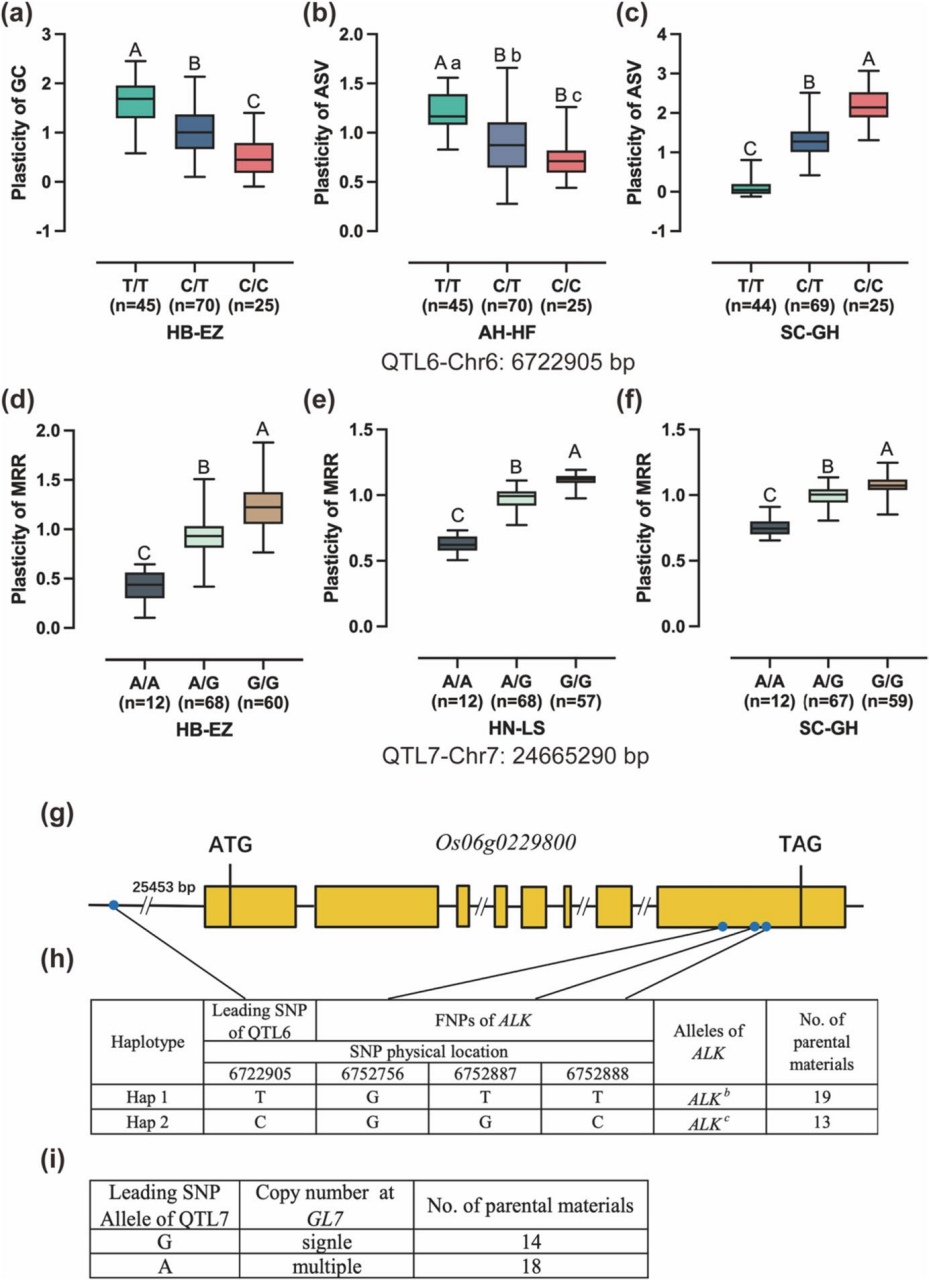
Genetic effects of QTLs and prediction of candidate genes. **a–f** Boxplots of plasticity of quality traits in hybrid combinations containing the different leading SNPs of QTLs. **a** Plasticity of GC in hybrid combinations containing the different leading SNPs of QTL6 in HB-EZ. **b–c** Plasticity of ASV in hybrid combinations containing the different leading SNPs of QTL6 in AH-HF and SC-GH. **d–f** The plasticity of MRR in hybrid combinations containing the different leading SNPs of QTL7 in HB-EZ, HN-LS, and SC-GH. Uppercase letters indicate statistically significant differences at *p* < 0.01; lowercase letters indicate statistically significant differences at *p* < 0.05. **g** The relationships between the QTL6 leading SNP and its candidate gene. **h** The haplotypes identified by combinations of QTL6 leading SNP and candidate gene FNPs. **i** The relationships between the QTL7 leading SNP and its candidate gene

We evaluated the genetic effects of all 13 plasticity QTLs (Fig. 6a–f, Fig. S2). Among these, two QTLs (QTL6 and QTL7) were detected in three trial locations, thereby establishing them as stable major QTLs for rice quality plasticity. As these two QTLs also demonstrated an effect on the BLUP measurement, further investigation into the genetic effects of these QTLs could lay a foundation for molecular breeding strategies aimed at enhancing both the quality and stability of hybrid rice.

The QTL6 was found to be associated with ASV and GC. The leading SNP (Chr6:6,722,905) of QTL6 had a − log10 (*p* value) of 7.83 in AH-HF, 13.61 in HB-EZ and 31.03 in SC-GH. Three genotypes of the leading SNP of QTL6 were identified. A significance analysis revealed that CC genotypes showed significantly lower GC plasticity compared to CT and TT genotypes in HB-EZ. It is worth noting that the direction of the genetic effects of the identical ASV plasticity allele varied across different cropping environments. In the AH-HF, CC genotypes exhibited significantly lower ASV plasticity compared to varieties with CT and TT genotypes. Conversely, in the SC-GH, CC genotypes showed significantly higher ASV plasticity compared to varieties with CT and TT genotypes (Fig. 6a–c). The QTL7 was associated with MRR. The leading SNP (Chr7:24,665,290) of QTL7 had a − log10 (*p* value) of 15.69 in HN-LS, 9.93 in HB-EZ, and 7.67 in SC-GH. The leading SNP alleles exhibited significantly different MRR plasticity, as shown in Fig. 6d–f. Notably, the AA genotypes displayed the lowest MRR plasticity value.

Within the associated genomic region of the QTL6 and QTL7, a total of 6 and 18 annotated genes were found, respectively. Among these genes, *ALK* and *GL7*, known for their functional annotations in controlling rice quality, were pinpointed as candidate genes for further analysis in rice quality plasticity. The *ALK* gene, located within QTL6, is a key gene controlling rice gelatinization temperature which encodes the soluble starch synthase II-3. The *GL7/GW7* gene, located within QTL7, encodes a protein that is homologous to the LONGIFOLIA proteins found in Arabidopsis thaliana. This protein is known to regulate cell elongation, thereby affecting grain length and grain shape.

## Discussion

Rice quality is one of the important goals in hybrid rice breeding improvement. The formation of rice grain quality is under genetic control and also influenced by the environment. Numerous studies have been undertaken to explore the impact of sowing dates on rice quality. However, due to differences in location, tested varieties, climatic conditions, and other factors, the outcomes of these studies lack consistency. Yao et al. observed that a delay in the sowing date leads to an improvement in the appearance quality of rice, but results in a decrease in the eating and cooking quality (Yao et al. 2011). Wang et al. found that a delay in the sowing date causes the CD, PGWC, AC, and GC to exhibit a declining trend (Wenting et al. 2021).

In this study, we analyzed the rice quality across multiple sowing dates in five trial regions located in Southern China and gathered the meteorological data for each region. It was observed that in the Yangtze River basin trials, the amount of high-quality rice (≥ grade 3 set by the Chinese Ministry of Agriculture) increases as the sowing date is delayed. The different sowing date essentially influences the weather conditions in rice growing. Previous studies indicated that an inappropriate sowing date will expose rice to unfavorable climatic conditions during the grain filling stage, resulting in a decline in rice quality (Cheng and Zhong 2001).

It has been proved that light, temperature, and rainfall during the grain filling stage are the pivotal climatic factors influencing rice quality (Resurreccion et al. 1977; Cheng and Zhu 1998). This study quantified and analyzed several crucial meteorological factors during the grain filling stage to assess their relationship with rice quality and endeavored to improve the precision of selecting the appropriate sowing date in high-quality hybrid rice production. Deng et al. found that the average daily temperature ranges of 22–27 °C in the grain filling stage are recommended to achieve high grain yield and quality for irrigated rice in the Yangtze River basin (Deng et al. 2015). Our results suggest that lower average daily temperature can contribute to improving the quality of rice, supporting the conclusion from Deng et al. In addition, we found that the impact of meteorological factors on rice quality exhibited varied trends among trial locations, and even a single factor may affect multiple quality traits in contrasting directions, resulting in a complex effect on overall rice quality. For instance, surface solar radiation was positively correlated with AC and negatively correlated with GC at HN-LS, while contrary correlation trends were observed at the other four trial locations. This difference may be due to the fact that HN-LS is located in the South China rice cropping region, with persistent high temperatures and abundant rainfall in the dry season, and has distinct climate conditions compared to the other trial locations within the Yangtze River basin. Simultaneously, the decomposed meteorological factors are unavoidably related in different ways. For instance, rainfall consistently results in a more significant temperature decrease within AH-HF, HN-CS, and HB-EZ in the middle and lower reaches of the Yangtze River basin in comparison to SC-GH and HN-LS. This discrepancy can be attributed to the lower altitude and the prevalence of hot and sunny weather in the summer. Concurrently, rain can reduce solar radiation, so SC-GH in the upper reaches of the Yangtze River has low solar radiation due to cloudy and rainy weather. As the quality of rice is the result of the combined effects of multiple meteorological factors, further analysis and modeling based on more experiments are necessary.

It is crucial to develop varieties with stable phenotypes that are less sensitive to environmental changes. This will help mitigate the adverse effects of frequent extreme climate events on rice yield and quality, guaranteeing the production of high-quality rice. This study employs phenotypic plasticity measurement to evaluate the stability of different quality traits in hybrid rice combinations. Combining ability analysis is a useful approach to selecting ideal parent lines for hybrid rice breeding (Chen et al. 2019). Through the analysis of combining ability in phenotype plasticity, we discovered that the TGMS lines ZXS, HX302S, and HY468S and the restorer lines HH8012, HH7503, DZ, LKSM13, HH2646, HH5106, HH8549, YZ, and WSSM534 showed low GCA effects on five or more quality traits plasticity across three rice cropping regions; they could be recommended for utilization in rice hybrid breeding programs to improve the stability of rice quality. A noteworthy result was found that parents with superior quality tend to exhibit low GCA values for plasticity and have more potential to develop a hybrid with optimal quality stability. ZXS is an *indica* TGMS line with high-quality approved in Hunan province in 2020. Its milled rice rate is 63.2%, chalky grains rate is 7%, chalkiness degree is 1.3%, amylose content is 16.1%, gel consistency is 60 mm, alkali digestion value is 7.0, and transparency is grade 1 (China Rice Data Center). A total of ten new hybrid rice varieties of ZXS have been nationally or provincially approved and certificated, and their quality has all reached the grade 2 or grade 1. The quality of ZhenLiangYouYuZhan (ZXS/YZ), a nationally approved new hybrid rice variety of ZXS, met grade-1 in the new variety regional trial and was awarded the Gold Award for Taste Evaluation of High-Quality Rice at the 4th National High-Quality Rice Competition in 2023. In our experimental field settings, the ZXS/YZ combination showed high-quality with grade 3 or superior performance in 12 out of 19 records, and these records occurred in all locations except for HB-EZ.

To uncover the genetic basis of phenotype plasticity in rice quality traits, GWAS for the quality plasticity was performed and identified 13 plastic QTLs. In line with previous studies (Zan and Carlborg 2020), we determined that plasticity is polygenic and exhibits a variable genetic basis for rice quality traits across regions. The genetic effects of plastic QTL can change in different locations. For instance, the QTL6 is a multi-effect QTL associated with the plasticity of ASV and GC, where genetic effects on ASV differ among trial locations. We also identified seven plastic QTLs overlapped in the GWAS of BLUP measurement of quality traits, indicating these QTLs could regulate the quality traits and respond to the plasticity of quality traits. This further validates previous research that there may be a connection between the genetic regulation of traits and plasticity (Zan and Carlborg 2020; Jin et al. 2023).

In the present study, we endeavored to identify the candidate genes of two major plasticity QTLs for rice quality. The region of these two major QTLs contains 24 annotated genes and 2 of them (*ALK* and *GL7*) are known to regulate the rice quality. *ALK* is the key gene controlling rice gelatinization temperature, which is closely associated with the eating and cooking quality in rice (Gao et al. 2011). According to previous studies, there are three main alleles of *ALK*, including *ALK*^*a*^, *ALK*^*b*^, and *ALK*^*c*^, with *ALK*^*c*^ controlling high gelatinization temperature, and *ALK*^*a*^ and *ALK*^*b*^ controlling low gelatinization temperature ( Chen et al. 2020; Huang et al. 2021). In this study, we identified two alleles of *ALK* including *ALK*^*b*^ and *ALK*^*c*^ in our parental rice accessions. Interestingly, we found that the leading SNP genotype of QTL6 was completely linked with *ALK*. Specifically, the CC genotype of the leading SNP of QTL6 was linked with *ALK*^*c*^ while the TT genotype was linked with *ALK*^*b*^ (Fig. 6g–h). Sequence analysis showed that among the above low plasticity GCA parents, all carried *ALK*^*b*^ except HH7503 and HH5106, which carried *ALK*^*c*^. To some extent, this result explains although rice varieties with low to medium GT are preferred by consumers, those carrying *ALK*^*c*^ alleles that regulate high GT are still commonly employed in rice breeding, potentially due to their plasticity levels.

The other plasticity candidate gene, the *GL7* locus, plays a significant role in grain size diversity and has been utilized in rice breeding. Wang et al. demonstrated that the copy number variation (CNV) at the *GL7* locus led to the difference in the rice grain size (Wang et al. 2015b). Whole genome sequencing analysis of parental rice accessions revealed that the CNV at *GL7* was linked with the leading SNP of QTL7. The varieties containing the CNV at *GL7* carry the AA genotype of the leading SNP of QTL7 and the remainder carry the GG genotype (Fig. 6i). Rice varieties with multi-copy at *GL7* locus exhibit lower plasticity in terms of milled rice ratio, which can be attributed to their long-grain phenotype. Further studies will be necessary to determine the effect of these candidate genes on phenotypic plasticity and their pleiotropic effects on quality traits and plasticity. These QTLs and candidate genes have the potential to contribute to improving rice quality and maintaining the stability of quality in breeding.

### Supplementary Information

Below is the link to the electronic supplementary material.

Supplementary file1 (DOCX 11485 KB)

Supplementary file2 (XLSX 366 KB)

## Data Availability

All data generated or analyzed during this study are included in this published article and its supplementary information files (Supplementary Table S1, S2, S3, S4, S5, S6, S7, S8, S9, S10; Fig. S1, S2).
