# Supplementary material for: Exploration of quality variation and stability of hybrid rice under multi-environments
Source: Mol Breed. 2024 Jan 15;44(1):4. doi: 10.1007/s11032-024-01442-3 (PMC10788329; doi:10.1007/s11032-024-01442-3)
Supplement: Supplementary file 1 — Supplementary file1 (DOCX 11485 KB) [file 11032_2024_1442_MOESM1_ESM.docx]

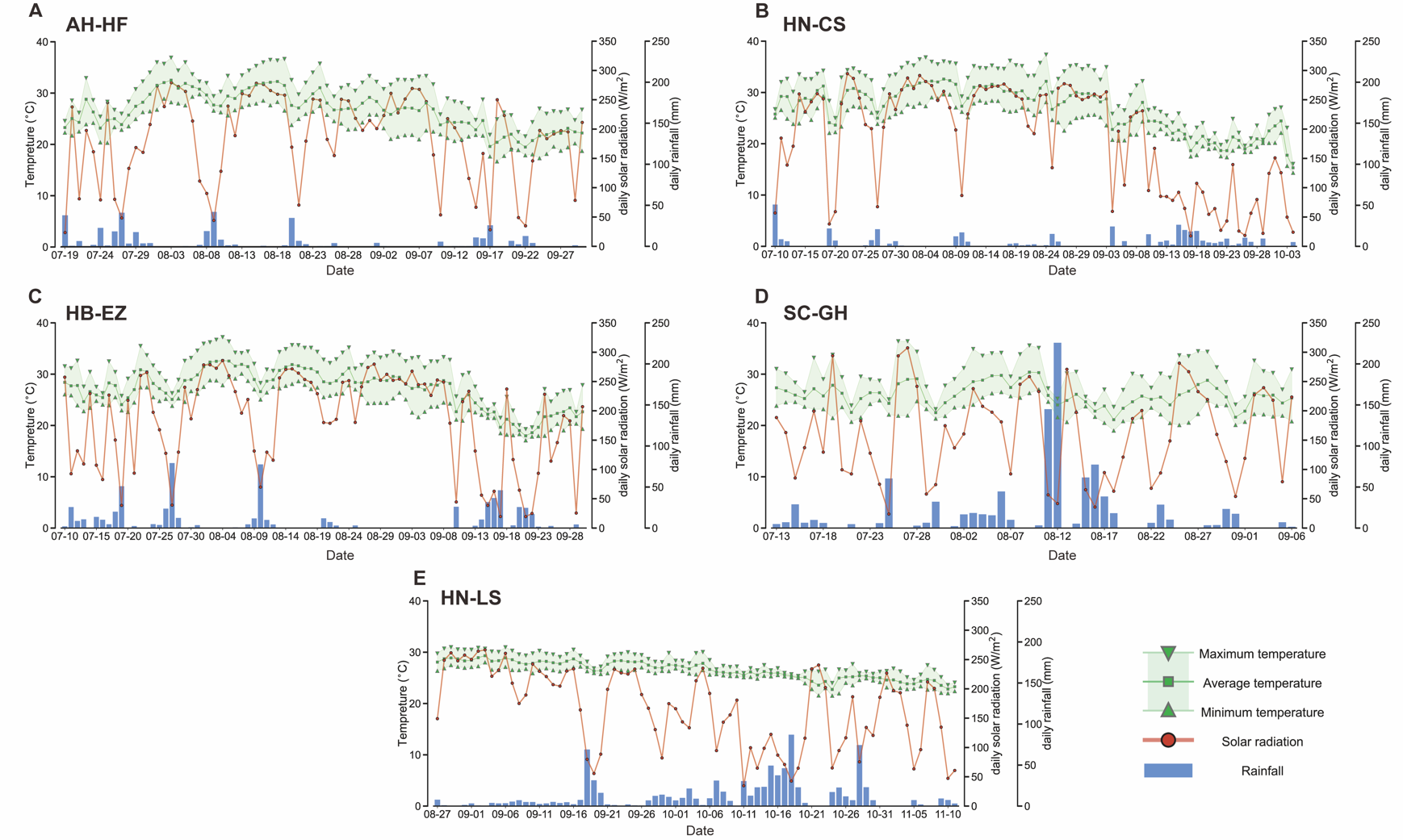


**Supplementary Fig. S1** Daily maximum temperature, average temperature, minimum temperature, solar radiation and rainfall at initial 15 days following the full heading across five trial locations.


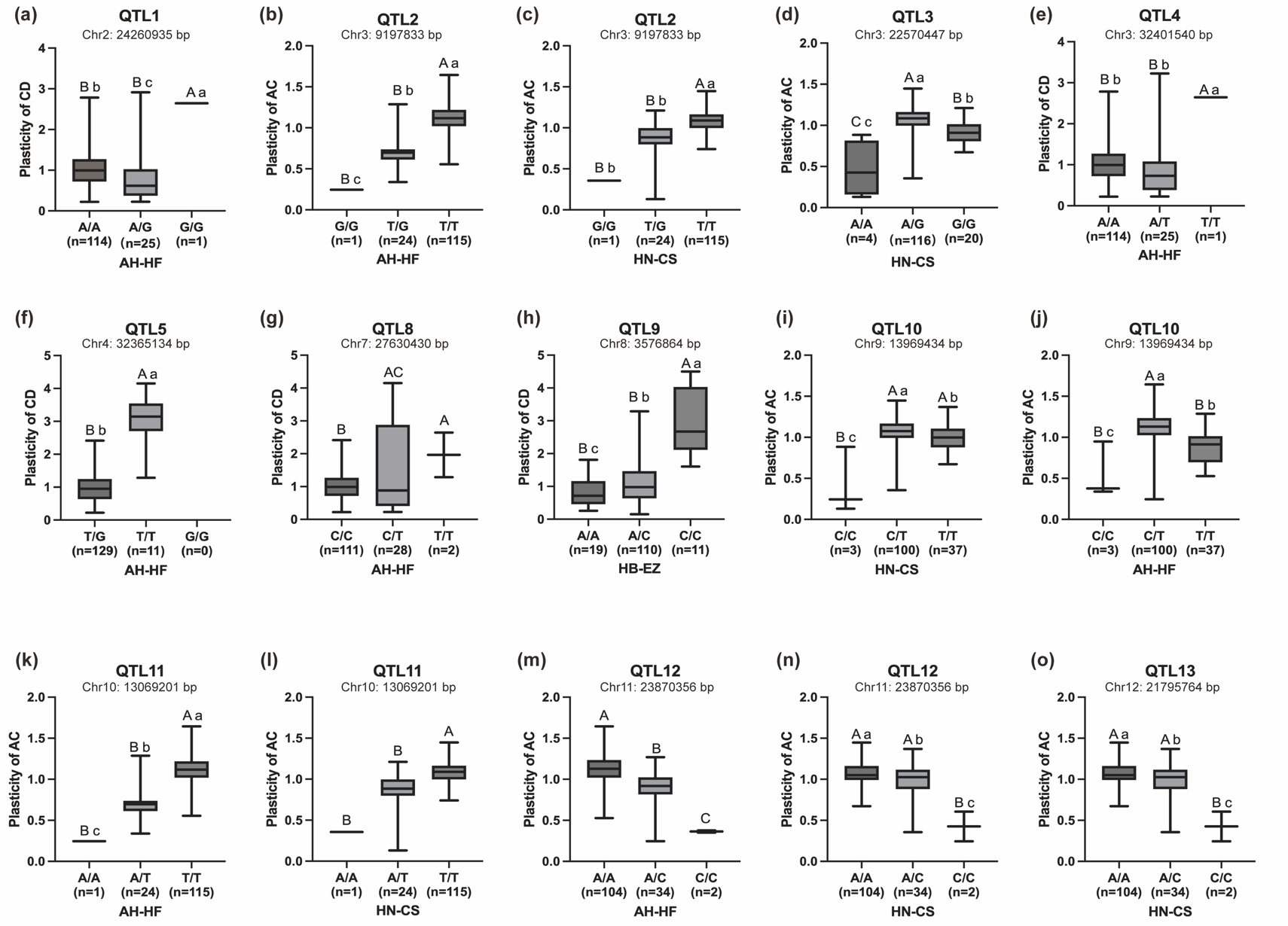


**Supplementary Fig. S2** Boxplots of plasticity of quality traits in hybrid combinations containing the different leading SNPs of QTLs. **(a)** The plasticity of CD in hybrid combinations containing the different leading SNPs of QTL1 in AH-HF. **(b-c)** The plasticity of AC in hybrid combinations containing the different leading SNPs of QTL2 in AH-HF and HN-CS. **(d)** The plasticity of AC in hybrid combinations containing the different leading SNPs of QTL3 in HN-CS. **(e)** The plasticity of CD in hybrid combinations containing the different leading SNPs of QTL4 in AH-HF. **(f)** The plasticity of CD in hybrid combinations containing the different leading SNPs of QTL5 in AH-HF. **(g)** The plasticity of CD in hybrid combinations containing the different leading SNPs of QTL8 in AH-HF. **(h)** The plasticity of CD in hybrid combinations containing the different leading SNPs of QTL9 in HB-EZ. **(i-j)** The plasticity of AC in hybrid combinations containing the different leading SNPs of QTL10 in HN-CS and AH-HF. **(k-l)** The plasticity of AC in hybrid combinations containing the different leading SNPs of QTL11 in AH-HF and HN-CS. **(m-n)** The plasticity of AC in hybrid combinations containing the different leading SNPs of QTL12 in AH-HF and HN-CS. **(o)** The plasticity of AC in hybrid combinations containing the different leading SNPs of QTL13 in HN-CS. Uppercase letters indicate statistically significant differences at p < 0.01, lowercase letters indicate statistically significant differences at p < 0.05.
